# How to boost the boosters? A survey-experiment on the effectiveness of different policies aimed at enhancing acceptance of a “Seasonal” vaccination against COVID-19

**DOI:** 10.1186/s13584-022-00536-7

**Published:** 2022-07-04

**Authors:** Talia Goren, Itai Beeri, Dana Rachel Vashdi

**Affiliations:** School of Political Sciences, University of Haifa, Mount Carmel, 31905 Haifa, Israel

**Keywords:** Vaccination acceptance, Vaccination policies, Incentives, Periodical vaccination, On-going pandemic

## Abstract

**Background:**

Evidence suggests a gradual decrease in the effectiveness of the anti-COVID-19 vaccines, stressing the potential need for periodical booster shots. However, it is hard to tell whether previously applied policies for enhancing vaccine acceptance will be as effective for repeated periodical booster shots during a pandemic. Hence, this study aims to explore the effectiveness of different health policies on periodical vaccination acceptance amidst an ongoing pandemic.

**Methods:**

A cross sectional online experiment was performed in a representative sample of 929 Israeli citizens. Participants were randomly allocated to 4 groups simulating different hypothetical periodical-vaccination-promoting policy scenarios: (1) Mandate (N = 229); (2) a negative monetary incentive (N = 244); (3) a positive monetary incentive (N = 228) and (4) information provision (N = 228). Compliance intentions and vaccine-acceptance-related variables were measured. Analysis included multivariate hierarchic logistic and linear regressions.

**Results:**

Compliance intentions levels were medium (M = 3.13 on a 1–5 scale). Only 20.2% of the sample demonstrated strong acceptance of periodical vaccination, which is lower than the acceptance rate of the seasonal flu shot in the country in the year preceding the pandemic. Type of policy was related to the extent to which a respondent strongly agreed to be periodically vaccinated or not. Specifically, strong acceptance was more likely when positive or negative incentives were presented in comparison to the mandate or information provision conditions. However, when examining the extent of compliance among respondents who were less decisive, the type of policy did not predict the extent to which these respondents intended to comply. In addition, compliance intentions were related with the perceived benefits and barriers of the vaccine, the perceived efficacy of getting vaccinated and social norms. Hesitator’s intentions were additionally associated with anti-COVID-19 vaccination history, perceived severity of the disease and trust in government.

**Conclusions:**

Pandemic-containing vaccines may be perceived as less effective and beneficial than pandemic-preventing vaccines. Individuals with different levels of motivation for periodical vaccination during a pandemic may be affected by different factors. While strongly opinionated individuals are affected by the type of vaccination-promoting policy, hesitators are affected by a larger number of factors, which provide policy makers with greater opportunities to enhance their vaccination intentions.

## Background

Two years after the outbreak of the Coronavirus pandemic, and at the midst of the Omicron variant outbreak, millions of people worldwide have taken Pfizer-BioNTech’s vaccine against the virus [1]. However, evidence suggests a decrease in the effectiveness of the vaccine over time, and against new variants of the virus [2–4]. Hence, the advantages and implications of an additional (fourth) booster shot are being investigated, while some countries have already begun offering it to immunocompromised populations [5, 6]. Preliminary evidence indicates that another shot will increases immunity against the virus [6], and some experts predict that much like vaccines against the seasonal flu, booster shots will be required periodically in order to maintain desired immunization levels against the Coronavirus [7, 8]. However, as the pandemic continues, governments find it harder to convince the public to get vaccinated and public acceptance of the booster shots seems to be decreasing [9, 10].

Aiming to enhance the COVID-19 vaccine acceptance rates, governments around the world are applying various policies for affecting individuals’ vaccination preferences, ranging from informative campaigns about the benefits of the vaccine to banned participation of un-vaccinated individuals in public events, cash rewards for those who get the shot and even mandatory vaccination [11–14]. These tactics, which represent different levels of government intrusiveness in individuals’ autonomous choice to get vaccinated [15, 16], may vary in their effectiveness on vaccination acceptance in the current pandemic and against other diseases [13, 17–19]. However, it is hard to tell whether such policies will be as effective for additional and periodical booster shots in the future, since repetitive adherence to health behaviors may require different incentives than one-time behaviors [20, 21].

One may think that the policies for increasing vaccine acceptance with periodical COVID vaccines, should be similar to those that were found effective in increasing annual seasonal flu vaccination compliance, since both require a repeated periodical behavior. However, neither the unique conditions and circumstances of an on-going global pandemic nor the public perceptions regarding the rapid development of a novel vaccine should be compared with those of routine, familiar and historically recommended flu vaccines. While the purpose of mass vaccination during an on-going pandemic is to contain it and prevent its further spread, vaccination against seasonal diseases is meant to prevent the initial outbreak of an epidemic [22–26]. In other words, vaccination during a raging pandemic, with possibly a newly developed vaccine, is performed mid-crisis, which inflicts great stress and uncertainties [27–31], while seasonal disease vaccination takes place as a preemptive measure to prevent crisis development, in a more controlled and relaxed context, using a well-known vaccine. While in both cases individuals’ goals may be to avoid the sickness the vaccines target, the conditions and considerations under which the choice to get vaccinated is made, may differ, consequently leading to different decisions. Indeed, recent evidence suggests that the unprecedented mental load and stressful conditions caused by the Coronavirus pandemic, may alter basic psychological mechanisms of appraisal and assessment of our surroundings [32, 33]. Furthermore, the speed in which a specific pandemic-fighting vaccine is developed and approved, may enhance public concerns regarding its safety, reliability and effectiveness [34, 35]. Hence, these very unusual circumstances may shape individuals’ decision-making processes regarding vaccine acceptance during an on-going pandemic. This study aims to explore the effectiveness of different health policies (i.e. neutral, positive and negative) for increasing periodical vaccination acceptance amidst an ongoing pandemic. Thus, this work suggests both theoretical and practical contributions. In addition to shedding light on the differences of incentivizing mechanisms of health behaviors in different contexts, it may provide policy makers and health authorities with practical insights for their upcoming challenge of periodical mass vaccination during an on-going global pandemic.

## Methods

### Design and setting

Aiming to investigate the effect of different policies on public willingness to get vaccinated periodically in the course of an on-going pandemic, we conducted an online survey-experiment, following the approval of the ethics committee of the Social Sciences Faculty of the University of Haifa (#292/20). The experiment measured participants’ intentions to get periodically vaccinated against COVID-19 upon presentation of one of 4 types of simulated policies that were meant to promote the adoption of this behavior: (1) a mandate; (2) a negative monetary incentive; (3) a positive monetary incentive and (4) information provision. We chose monetary incentives and not other types of non-monetary incentives for several reasons. First, we were interested in measuring the effect of incentive valence (i.e., positive “rewards” vs. negative “penalties”), and not the effect of incentive type, in terms of monetary vs. non-monetary. Hence, using monetary incentives allowed us to control for the value of the incentives and simulate equal incentives with different valence. Second, several months prior to our study the Israeli government had already applied non-monetary incentives in respect to the anti-COVID vaccine (e.g., the “Green Pass”), including for those receiving the third (booster) shot. Hence, their effect on periodical vaccination was already publicly apparent. Therefore, our study focused on investigating the effect of incentive types that were not yet applied.

The survey was conducted on the 8th-12th of November 2021, during which the number of severely ill patients in Israel was relatively low,^1^ the campaign for the booster (third) shot was on the go and the shot was available for the general population for over 2 months [36]. Data collection was performed prior to the outbreak of the Omicron variant and therefore preceded any public discussions regarding the possibility of recommending a fourth booster shot. Nonetheless, irresolute expectations of public health experts regarding the possible necessity of a periodical renewal of the vaccine were already made public in Israel since the beginning of the national vaccination campaign [37–39]. These circumstances made the notion of a policy that promotes periodical vaccination feasible.

### Sample

929 adult participants were recruited via a survey company and were rewarded for their time and cooperation about 5$ (USD; ~20 NIS). They composed a representative sample of the Israeli population with regards to gender, age, residence area and sector (including the Arab and Ultra-Orthodox communities). See Table 1 for the demographic features of the overall sample and the demographic features of the sectors.

**Table 1:** Means and standard deviations of the demographic variables in the overall sample and by sector.

	Overall sample			General Jewish N = 757*			Arab N = 108*			Ultra-Orthodox N = 55*		
	N	Mean	Std	N	Mean	Std	N	Mean	Std	N	Mean	Std
Gender^1^	924	.48	.500	755	.49	.500	107	.42	.496	55	.44	.501
Age^2^	929	39.45	14.043	757	40.46	14.255	108	35.63	11.897	55	33.27	12.409
Education^3^	920	4.09	1.320	753	4.11	1.330	104	4.27	1.192	54	3.56	1.341
Parenthood^4^	914	.43	.496	751	.41	.492	104	.48	.502	54	.70	.461
Income^5^	841	2.39	1.155	681	2.54	1.138	103	1.75	1.064	50	1.74	.853

*The sample included 9 additional participants that did not report their sectorial affiliation and hence were excluded from any sector-involving analysis; ^1^Gender: 1 = male, 0 = female; ^2^Age in years; ^3^Education: a 6-point scale ranging from elementary school = 1 to a masters' degree and beyond = 6; ^4^Parenthood: 1 = "has children", 0 = "does not have children"; ^5^Income level: relative to average household income: ranging from 1 = "much lower" to 5 = "much higher"

### Procedure

After providing informed consent, participants were randomly allocated to one of 4 experimental cells which represented 4 types of government intervention policies aimed at promoting health behavior adoption [15]. In each cell, participants were presented with one vignette showing one type of policy as a transcribed simulated radio news report. The first 3 cells simulated newly issued government policies, which were decided upon “last night”, based on recent studies that indicated the need to renew the COVID-19 vaccine every 6 months in order to maintain sufficient immunization levels: (1) a new law mandating vaccination of adults against COVID-19 every 6 months (no specific penalties for violators were mentioned); (2) a negative incentive: a new regulation increasing health taxes for those who will not take the shot every 6 months by 50%; (3) a positive incentive: a new regulation according to which health taxes for those who will get vaccinated every 6 months will be decreased by 50%. In Israel, health taxes are relative to ones’ income. Hence, by introducing monetary incentives that depend on one’s health taxes, we avoided any relativity bias effects that absolute monetary incentives may induce, given variance in income levels and financial point of reference [40]. The fourth cell simulated information provision regarding a new study performed by researchers in the Israeli Ministry of Health, which concluded that a booster shot is needed every 6 months in order to maintain sufficient immunization levels.

Next, participants were asked if they have any medical issues preventing them from getting the anti-COVID-19 vaccine on doctors’ orders. Those who responded “yes” were excluded from the analysis. Then, the participants were asked to state their intention level to get vaccinated every 6 months. This was followed by questions measuring additional variables that are well-known for potentially affecting vaccine acceptance, such as previous vaccination against the disease, perceptions regarding the threat posed by the Coronavirus, the benefits and barriers of getting the shot periodically and one’s efficacy of getting the shot [41–43]. We also measured levels of perceived social norms for performing the advised behavior and trust in government, which are renowned factors of compliance with government instructions [44–46]. In addition, we asked participants to indicate the recovery status from the COVID-19 virus of themselves and their close relatives, as we assumed they may impact vaccination inclinations. The survey was concluded with demographic questions which were followed by a debriefing that stressed that the presented instructions and information were bogus.

### Measures

#### Dependent variable

*Compliance intentions* were measured using the protocol for measuring behavioral intentions in a medical context [47], which is composed of three 5-point items, each presenting a different verb regarding intentions: “I expect/ want/ intend to get vaccinated against COVID-19 every 6 months”; (1="strongly disagree” to 5="strongly agree”). An average score was calculated following an inner reliability check (α = 0.942), and constituted our intention to comply variable.

#### Independent variable

*Policy type* was defined by the type of government intervention in one’s autonomy to make choices regarding vaccination, presented to the participant. We adapted our vignettes to 4 levels of the Nuffield Council on Bioethics’ [15] intervention ladder for government actions for promoting health behaviors. The ladder ranges from low to high intervention levels in the following steps: 1="Do nothing”; 2="Provide information”; 3="Enable choice”; 4="Guide choices through changing the default policy”; 5="Guide choices through incentives”; 6="Guide choice through disincentives”; 7="Restrict choice” and 8="Eliminate choice (mandatory regulation)”. For this experiment we simulated government policies corresponding to steps 2,5,6,8 (information provision about the advantages of getting vaccinated every 6 months, positive incentives for those who will get vaccinated every 6 months, negative incentives for those who will not get vaccinated every 6 months and elimination of choice by presenting a mandatory vaccination every 6 months), as they represent the relevant spectrum of the ladder and match the context of this study.

#### Control variables

*Previous vaccination against COVID-19* was measured by a single YES/NO item in which participants were asked whether they had received the COVID-19 vaccination in the past.

*Perceived benefits* of the promoted behavior was measured by asking participants to state their level of agreement with the following 2 items [48, 49]: (1) “Getting vaccinated against COVID-19 every 6 months is a good idea because it makes me feel less worried about catching COVID-19”; (2) “Getting vaccinated against COVID-19 every 6 months will reduce my chances of catching COVID-19”. Answers could range between 1="strongly disagree” to 5="strongly agree”. These items were highly correlated (Pearson’s’ r = 0.853; p < 0.01) and hence averaged to a single 1–5 scale.

*Perceived Barriers*: since safety barriers are considered the most prominent barriers for vaccinations [50], we measured perceptions regarding the safety of the vaccine, with two 5-point items, used in previous work on health behavior adoption [51]. Example item: “I have concerns about the safely of getting the COVID-19 vaccination every 6 months”. Answers could range between 1="strongly disagree” to 5="strongly agree”. The items were highly correlated (r = 0.74; p < 0.01) and averaged to a single 1–5 scale.

*Perceived threat* posed by the disease was measured by two dimensions often used in health behavior research in general and in vaccine compliance research in particular [e.g., 52]: (1) *Perceived Severity* of the disease and the (2) *Perceived Susceptibility* of becoming ill with it. These were measured by three items each, using Lin et al’s [49] items for vaccination intentions during the Coronavirus pandemic. Example item for severity is: “I will be very sick if I get COVID-19”; Example item for susceptibility is: “Getting COVID-19 is currently a possibility for me”. Answers could range between 1="strongly disagree” to 5="strongly agree”, and averaged to a 1–5 scale for each of the variables (α = 0.71(severity) and α = 0.745 (susceptibility)).

Inspired by previous work on perceived self-efficacy for complying with health measures during the current pandemic [48, 53], we measured *self-efficacy for preforming the promoted behavior* using 3 items. Example item is “I am able to get vaccinated against COVID-19 every 6 months if I want to”. Answers could range from 1="strongly disagree” to 5="strongly agree” and averaged to a 1–5 scale (α = 0.827).

*Trust in government* was measured with 5 items based on the cynicism/trust in government scale [54, 55]. Example item: “How much of the time do you think you can trust the government to do the right thing?” (0-“never” to 100- “always”). An averaged 1–5 scale was created (α = 0.895).

*Social norms* of compliance with the promoted behavior: Inspired by Scholz and Brook [56], we used a single item in which participants were asked to assess the rate to which their close friends and relatives will get vaccinated every 6 months, ranging from 0="no one” to 100="everyone”.

*Recovery status from COVID-19* of the participant and his/her relatives was measured by the following two items: “were you/ your close relatives sick with COVID-19 in the past?“. Participants could reply “Yes”/ “No” or decline to respond. Decliners were removed from all analyses involving this variable.

*Demographic variables*: *Gender; Age* (in years); *Parenthood* (1="has children”, 0="does not have children”); *Income*: A 5-point scale, relative to average household income: 1="much lower” to 5="much higher”; *Education*: A 6-point scale ranging from 1 = elementary school to 6 = a masters’ degree and beyond.

### Statistical analysis

Using SPSS27, we conducted an initial descriptive statistical analysis, followed by a multivariate logistic regression analysis and a multivariate linear regression analysis.

## Results

### Compliance intentions

**Fig. 1:**
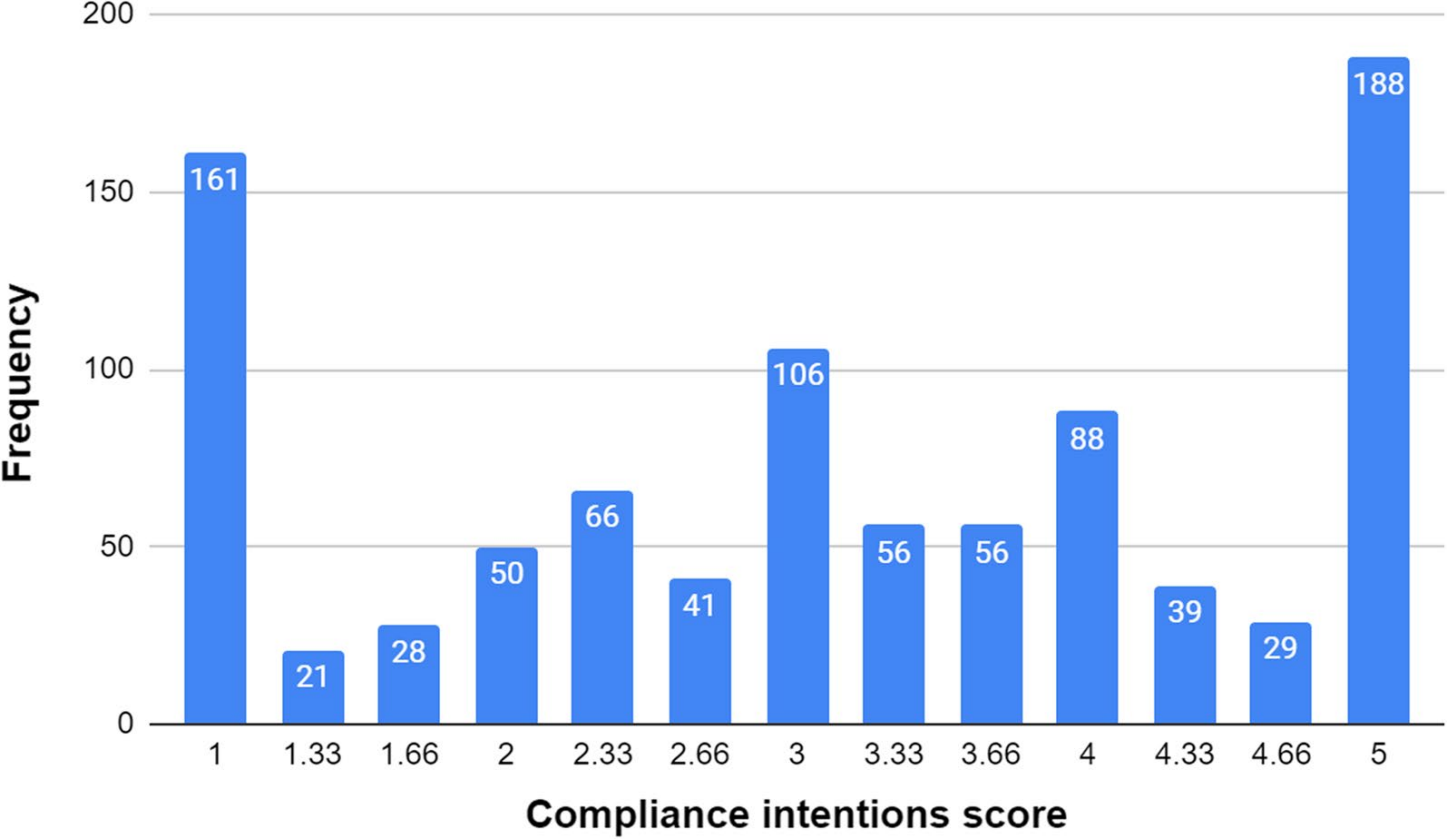
Distribution of intentions to get vaccinated every 6 months. N = 929; Mean = 3.13; SD = 1.41

The mean score of the intentions to get periodically vaccinated against COVID-19 was 3.13 (SD = 1.41; on a 1–5 scale), indicating an overall moderate level of acceptance. However, a close examination of the distribution of the data revealed a more complex picture. As Fig. 1 shows, a significant percentage of the participants reported their periodical vaccination intentions to be either extremely high (i.e., 5; 20.2%) or nonexistent (i.e., 1; 17.3%), while the rest of the sample (62.5%) seems to be normally distributed across the scale between them. This result prevented us from preforming parametric analysis of this variable, but more importantly, it indicated that in the case of our sample, vaccination acceptance should be viewed as a categorical variable rather than a continuous one. Following this logic, we grouped our participants into 3 groups: (1) *Strong accepters*—those whose compliance intentions’ score was 5, indicating the maximal positive intention to get vaccinated (20.2%); (2) *Hesitators*—those whose compliance intentions’ score ranged between the minimum and maximum possible scores, i.e., 1.33 and 4.66 (62.5%); and (3) *Strong opposers*—those whose compliance intentions’ score was 1, indicating no intention to get vaccinated at all (17.3%).

## Vaccination acceptance by policy type

**Fig. 2:**
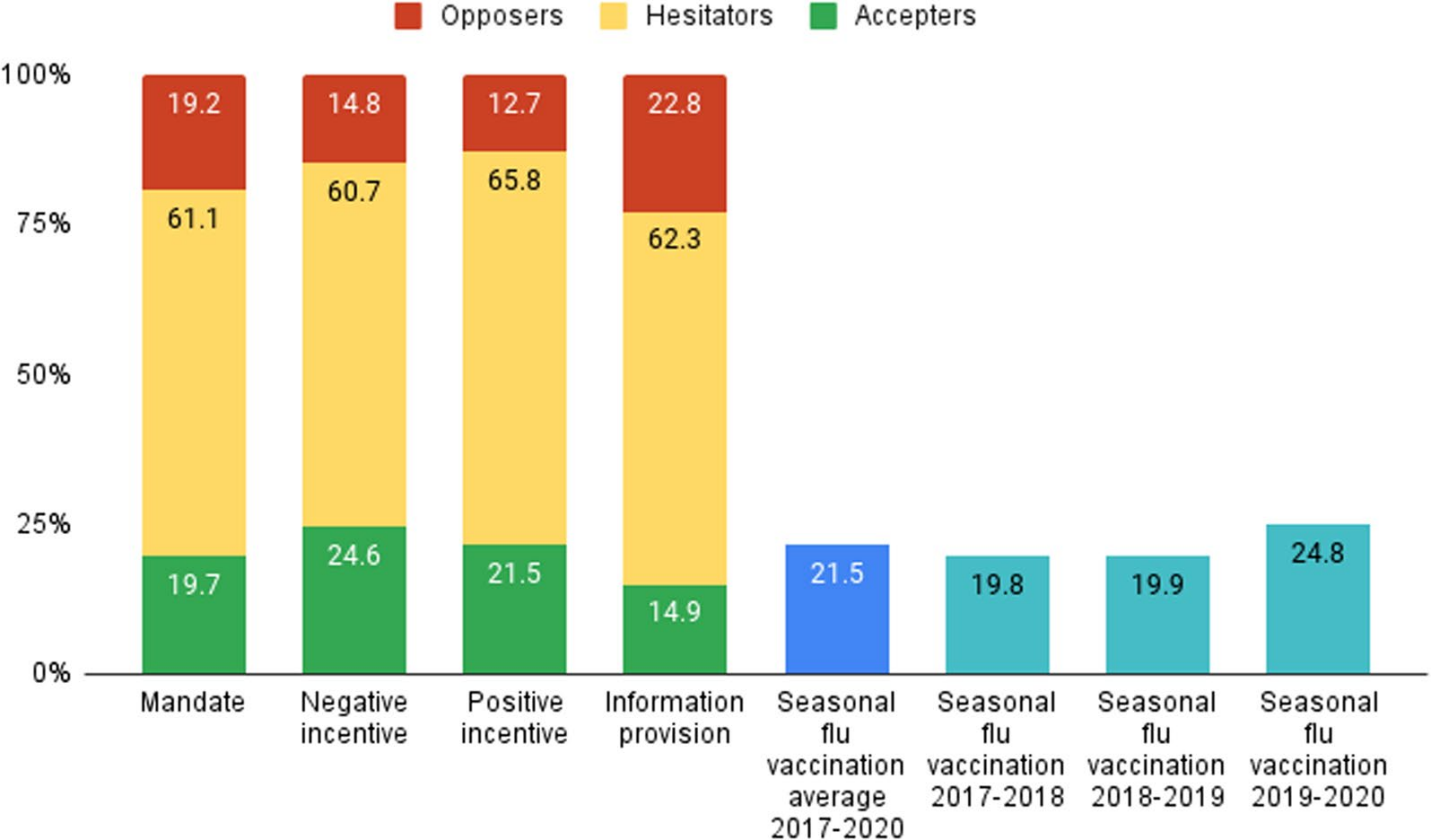
Distribution of vaccination accepters, hesitators and opposers by policy type and vaccination rates against seasonal flu in Israel in the three winters preceding the Coronavirus pandemic. Mandate-N = 229; Negative incentive-N = 244; Positive incentive-N = 228; Information-N = 228; Seasonal vaccination rates data: Official report of the Israeli Ministry of Health: https://www.health.gov.il/PublicationsFiles/Flu2019_2020.pdf

The highest rate of strong-accepters was registered in the negative incentive group (24.6%). This was followed by the positive incentive group (21.5%), the mandate group (19.7%) and the information provision group (14.9%; see Fig. 2). Interestingly, the mirror picture of this order was not identical for strong opposers. While the information provision group did have the highest rate of opposers (22.8%), it was followed by the mandate group (19.2%), then by the negative incentive group (14.8%) and by the positive incentive group (12.7%). In other words, while the information provision policy yielded the lowest acceptance rate and the highest opposition rate, the negative incentive policy yielded the highest acceptance rate but not the lowest opposition rate.

In order to investigate the significance of these differences in vaccination intentions of strong accepters between vaccination policies, above and beyond other vaccination acceptance enhancing variables, we performed a hierarchic logistic regression, in which the dependent variable was either strong accepters or others. We ran 3 models. Model 1 included demographic variables. In Model 2 we added other variables previously found to be associated with vaccination acceptance, and Model 3 included the type of policy, represented by dummy variables with negative incentive as a reference group. As Table 2 shows, the type of policy did have a significant effect on periodical vaccination intentions above and beyond the other factors. The mandate and the information provision policies had a significantly weaker effect than the negative incentive. But the positive incentive’s effect was not significantly weaker than the negative incentive’s effect. We ran the same model with information provision as the reference group and the results were similar. That is, both negative and positive incentives were significantly different than information provision (b = 1.11; SE = 0.33; p < 0.01 and b = 0.77; SE = 0.34; p < 0.05, respectively), but the mandate condition was not. Simply put, both types of incentives were more effective in enhancing the chances of vaccination acceptance, compared to information provision and a general mandatory directive.

**Table 2:** Logistic regression with periodical vaccination strong-acceptance as a dependent variable

	Model 1			Model 2			Model 3		
	B	SE	Exp(B)	B	SE	Exp(B)	B	SE	Exp(B)
Constant	−2.446***	0.397	0.087	−5.901***	1.362	0.003	−5.756***	1.406	0.003
Gender	0.181	0.176	1.198	− 0.271	0.241	1.011	− 0.246	0.245	0.782
Age	0.028***	0.006	1.028	0.011	0.008	1.022	0.012	0.008	1.012
Parenthood	− 0.132	0.183	0.876	0.022	0.242	1.000	0.027	0.247	1.027
Income	0.128	0.080	1.137	0.000	0.105	0.875	− 0.014	0.107	0.986
Education	− 0.087	0.069	0.916	− 0.134	0.091	1.280	− 0.148	0.092	0.862
Sector-UO^ab^	0.047	0.420	1.048	0.247	0.630	2.107	0.089	0.634	1.093
Sector-Arab^b^	0.147	0.288	1.158	0.745	0.420	0.770	0.716	0.434	2.047
Past COVID vaccination				− 0.261	0.889	2.662	− 0.248	0.925	0.780
Perceived benefits				0.979***	0.176	0.401	1.027***	0.180	2.793
Perceived barriers				− 0.914***	0.144	1.049	− 0.912***	0.146	0.402
Severity				0.048	0.160	1.382	0.077	0.163	1.080
Susceptibility				0.324*	0.171	1.364	0.280	0.173	1.323
Self-efficacy				0.311*	0.176	0.995	0.311*	0.179	1.365
Trust in Government				− 0.005	0.005	1.021	− 0.005	0.005	0.995
Social norms				0.021**	0.007	1.053	0.023**	0.007	1.023
Had COVID				0.052	0.389	0.917	0.126	0.389	1.134
Relatives had COVID				− 0.087	0.258	1.011	− 0.033	0.262	0.967
Mandate v. negative incentive							− 0.694*	0.324	0.500
Positive Incentive v. negative incentive							− 0.340	0.321	0.712
Information v. negative incentive							−1.113**	0.332	0.328
-2 Log likelihood	809.810			495.106			482.395		
Nagelkerke R^2^	0.052			0.537			0.552		

N = 799; *p < 0.05; **p < 0.01; ***p < 0.001; ^a^UO—Ultra-orthodox; ^b^Reference group: General Jewish population

Furthermore, several other factors were found to be associated with periodical vaccination acceptance above and beyond the type of policy: Perceived benefits and barriers of periodical COVID-19 vaccination (b = 1.03; SE = 0.18; p < 0.001 and b=-0.91; SE = 0.15; p < 0.001, respectively), perceived efficacy to get vaccinated (b = 0.31; SE = 0.18; p < 0.05) and social norms (b = 0.02; SE = 0.07; p < 0.01). However, contrary to previous evidence regarding vaccination intentions, past vaccination against COVID-19, the perceived threat posed by the disease (severity and susceptibility) and trust in government did not predict the likelihood of periodical vaccination acceptance (though susceptibility was found to be associated with acceptance in model 2, when the type of the policy was not included in the analysis). Having COVID-19 in the past or having relatives who were sick in the past were not associated with compliance intentions either.

## Hesitators’ vaccination intentions

Since the compliance intentions of the hesitators were normally distributed as a continuous variable, we performed a multiple OLS hierarchic regression analysis, aiming to examine if the type of policy influences one’s intention to comply within this group. We ran the same 3 models as the ones performed in the accepters’ analysis. As Table 3 shows, unlike the case of strong accepters, for hesitators the type of policy did not have a significant effect on the extent to which they intended to comply. However, several other factors did (model 3): Female hesitators were more likely to have higher vaccination intentions than male hesitators (b=-0.15; SE = 0.06; p < 0.05); Perceived benefits and barriers for periodical COVID-19 vaccination (b = 0.25; SE = 0.03; p < 0.001 and b=-0.26; SE = 0.03; p < 0.001, respectively), the perceived severity of the disease (b = 0.09; SE = 0.04; p < 0.05), efficacy to get vaccinated (b = 0.15; SE = 0.04; p < 0.001), trust in government (b = 0.005; SE = 0.001; p < 0.001) and social norms (b = 0.003; SE = 0.001; p < 0.05) were all significantly associated with compliance intentions. However, perceived susceptibility for contracting the disease, previous vaccination against COVID and COVID recovery status of the participants and their relatives did not predict intentions for periodical vaccination among hesitators.

**Table 3:** Results of an OLS regression analysis of hesitators’ periodical vaccination intentions

	Model 1		Model 2		Model 3	
	B	S.E.	B	S.E.	B	S.E.
Constant	2.515***	0.181	1.422***	0.268	1.438***	0.272
Gender	0.015	0.080	− 0.115*	0.058	− 0.115*	0.058
Age	0.004	0.003	− 0.001	0.002	0.000	0.002
Parenthood	− 0.065	0.084	0.036	0.059	0.036	0.059
Income	0.104**	0.038	− 0.014	0.026	− 0.013	0.027
Education	0.054	0.032	0.033	0.022	0.033	0.022
Sector-UO^ab^	0.083	0.188	0.205	0.134	0.197	0.136
Sector-Arab^b^	− 0.039	0.144	0.087	0.104	0.087	0.105
Past COVID vaccination			0.290*	0.139	0.283*	0.140
Perceived benefits			0.245***	0.034	0.248***	0.034
Perceived barriers			− 0.256***	0.034	− 0.256***	0.034
Severity			0.082*	0.041	0.085*	0.042
Susceptibility			0.064	0.041	0.059	0.041
Self-efficacy			0.154***	0.037	0.155***	0.037
Trust in Government			0.005***	0.001	0.005***	0.001
Social norms			0.003*	0.001	0.003*	0.001
Had COVID			− 0.078	0.089	− 0.076	0.089
Relatives had COVID			0.007	0.061	0.009	0.062
Mandate v. negative incentive					− 0.040	0.079
Positive Incentive v. negative incentive					− 0.015	0.077
Information v. negative incentive					− 0.050	0.080
R^2^│ΔR^2^	0.34	0.34*	0.555	0.522***	0.538	0.000

N = 491; *p < 0.05; **p < 0.01; ***p < 0.001; ^a^UO—Ultra-orthodox; ^b^Reference group: General Jewish population

These results suggest that acceptance chances are not affected by the same factors when comparing strong accepters’ and hesitators’. While both groups’ vaccination intentions seem to share similar associations with perceived benefits, barriers, one’s self-efficacy to get periodically vaccinated and social norms, hesitators’ intentions seem to be additionally affected by COVID-19 vaccination history, the perceived severity of the disease, and trust in government. Interestingly, while hesitators’ intentions are not affected by either type of policy applied in order to enhance vaccination, acceptance rates among strong accepters are associated with the type of policy. More specifically, they are positively affected by both types of incentives and by negative incentives to a slightly greater extent.

## Discussion

Our study shows that more than a third of the Israeli population has strong opinions—either positive or negative—regarding periodical vaccination in an on-going pandemic, while the great majority, about 60%, hesitates. Previous studies on vaccine hesitancy regarding the COVID-19 non-periodical vaccine in Israel and in other countries report much lower levels of hesitancy [25, 57]. This stresses the conceptual and practical difference in public acceptance of periodical and non-periodical vaccines during a pandemic. As described above, the overall strong-acceptance rate for getting a vaccine every six months was 20%. This rate is lower by 5% from the actual vaccination rate against seasonal flu in the country, as registered in the winter prior to the Corona pandemic (2019/2020: 24.8%), and about 1.5% lower than the average seasonal flu vaccination rates in the three preceding winters in the country (2017–2020: 21.5%).^2^This suggests that periodical vaccination during an on-going pandemic may be perceived as less reliable and effective for preventing one’s contraction, compared to pandemic-*preventing* vaccinations (e.g. seasonal flu). One may argue that vaccination intentions regarding newly and rapidly developed vaccines such as the COVID-19 vaccine should not be compared with acceptance rates of well-known and practiced seasonal vaccines, such as the flu shot. However, the latter is the only vaccine that is recommended to the Israeli public by the ministry of health and health officials to be taken regularly and periodically. Hence, it is the most suitable, yet surely not perfect, reference.

The results demonstrate that for people with strong opinions about periodical vaccination, positive and negative monetary incentives may enhance periodical vaccination acceptance during an ongoing pandemic to a greater extent than mandates or information provision. Importantly, this implies that during an ongoing pandemic soft health policies, such as nudges and information provision [58], may not suffice for achieving the desired vaccination acceptance rate, while harder “carrot and stick” policies are likely to be more effective. These results coincide with evidence regarding the effectiveness of incentives for enhancing vaccination and other health behaviors [59–61]. However, the effect of incentives may be limited to vaccination acceptance of people with strong opinions regarding periodical vaccination, as they do not seem to affect those of hesitators. This may be explained by the varying effect of incentives on differently motivated individuals. The behavioral economics literature suggests that levels of motivation, i.e., one’s preliminary willingness to carry out a specific action, may shape the way a person is affected by external incentives offered to him/her in the attempt to further enhance their motivation to perform the action [62–64]. Evidence shows, that though usually highly-motivated individuals are less affected by external incentives [65, 66], in some cases, highly motivated persons can be more sensitive to monetary incentives than others [67].

Though some variables seem not to affect neither hesitators’ nor accepters’ vaccination intentions (e.g., perceived susceptibility for contracting the disease, one self’s and one’s relatives’ recovery status and demographic variables), hesitators and acceptors differ in the variables that are associated with their vaccination intentions. Hesitators’ vaccination intentions, compared to accepters’ vaccination intentions, are affected by a larger number of factors. Aside from the periodical vaccination benefits, barriers, social norms and perceived self-efficacy, hesitators’ intentions are also affected by their own vaccination history against the current pandemic-generating virus, the severity they attribute to the disease, and the level of trust they have in their government. This variance may not be surprising as the psychological literature suggests that strong opinions are less likely to be changed [68]. In other words, individuals who have yet to decide regarding their actions, or in our context—hesitators, are prone to be easily influenced by multiple factors, compared to people who have already made up their mind. This insight may serve a key concept in enhancing periodical vaccine acceptance during an ongoing pandemic, as it stresses the multiple paths through which hesitators—who compose the largest group in the population—can be convinced to get vaccinated.

It is worth noting that one’s perceived susceptibility for contracting the disease (i.e., perceptions regarding one’s likelihood of being infected with it) did not affect periodical vaccination intentions in neither of the examined groups. This result may seem as somewhat unintuitive and does not align with later information regarding high vaccination rates of the third and fourth doses among people who are at high risk of contracting the disease and/or being severely affected by the virus [10]. However, research indicates that the psychological and behavioral mechanisms that enable the performance of a one-time behavior, even if it is not the first time it is being performed, and performing a repeated behavior periodically, are different [69–71]. This means that people who agree to take a specific additional shot (given a specific state of the pandemic), would not necessarily agree in advance to get a shot periodically (when future conditions and severity of the pandemic are unknown) and vice versa.

### Practice implications

To enhance vaccination acceptance during a pandemic, based on the current study, policy makers should not make do with soft policies such as informative campaigns about the benefits and safety of the vaccine, but rather accompany them with monetary incentives. Both positive (“rewards”) and negative (“penalties”) incentives will enhance vaccination acceptance to a greater extent than either mandates or simply providing pro-vaccine information. In addition, vaccine hesitancy may be reduced by stressing the ease and simplicity in getting the vaccine and the severity of the disease in unvaccinated individuals. Furthermore, policy makers and health officials should take measures to preserve and strengthen public trust in the government, as it may contribute to higher vaccination intentions among hesitators.

While retrospect data regarding high vaccination rates of the fourth dose among people in high-risk groups may lead to the assumption that higher coverage rates can be achieved when periodical vaccines are offered to the public as separate doses rather than routine periodical ones, at least among more sensitive groups, this strategy may be impractical and even destructive and unethical. If and when it is agreed upon public health professionals that a routine periodical vaccine is needed, offering the public each dose of the vaccine separately, without providing full information about the necessity of renewing the vaccine periodically, will damage public health professionals’ reliability and mislead the public. Hence, offering ad-hoc vaccines when the importance of routine and periodical ones is already realized, should be avoided.

### Study limitations

The study was performed in Israel, hence, findings should be treated with caution as they may not represent other countries and cultures [72]. Moreover, our experiment simulated a semi-annum vaccination requirement, which challenges our findings’ applicability for other longer or shorter time spans. Furthermore, our study focuses on a single type of monetary incentive with a specific value, relative to the participant’s value of health taxes. Therefore, the results may not apply to other types of incentives, both monetary and non-monetary. In addition, the use of intentions may not fully predict actual vaccination behavior. However, robust behavioral theories, as well as many vaccination acceptance-focused studies consider vaccination intentions as a good indicator of future behavior [73–78]. Furthermore, the fact that the policies presented to the participants were hypothetical is an additional limitation, as they could have been perceived by the participants as not plausible. However, considering the novelty of various health guidelines issued by health authorities and unprecedented policies applied by the government during the COVID pandemic [79], even hypothetical policies scenarios as the ones we presented to our participants may seem authentic and realistic. In addition, the presented scenarios were somewhat plausible, considering the public discussion and media coverage regarding the possible need for a periodical vaccination and various public suggestions regarding vaccination incentivization, which were similar to the ones presented in our study [80–82]. Another limitation is the cross-sectional design of this study. Cross-sectional studies capture a “snapshot” of the examined population, at a specific time and under specific circumstances [83]. Therefore, it is possible that under different conditions our results would have been different. More specifically, it is possible that if a similar study would have been conducted under a severer pandemic status, its results would have been different. However, our survey included a measure of perceived susceptibility of contracting the virus as a control variable. This variable can be considered a proxy for the status of the pandemic, as it reflects perceptions of contagion chances which are usually based, to some extent, on the objective severity of the pandemic [84, 85]. Given that our analysis indicated this variable did not have a significant effect on vaccination intentions, it is possible that the severity of the pandemic may not have a dominant effect on compliance intentions and on our results.

## Conclusions

This study sheds light on the factors and health policies that may affect periodical vaccination acceptance during an ongoing pandemic. First, it indicates that individuals with different levels of motivation for periodical vaccination may be affected by different factors. Particularly, hesitators, compared to strong accepters, are affected by a larger number of factors, which provide policy makers and health officials with greater opportunities to enhance the likelihood of their vaccination.

Second, our study suggests that vaccination policies make a difference for people with high vaccination motivation, but they do not affect vaccine hesitators. More specifically, it shows that information provision and mandating directives alone are least effective for enhancing vaccination acceptance. Monetary incentivizing policies, however, may increase vaccination acceptance to a greater extent. Nevertheless, even when the most effective policy is applied (i.e., a negative incentive), the willingness for a periodical vaccination amidst a pandemic, is just slightly higher compared to the average vaccination rate for a pandemic-preventing *unincentivized* vaccination (i.e., the seasonal flu shot). In other words, during a pandemic it takes incentives in order to reach similar average acceptance rates as those of recommended and un-incentivized vaccines against seasonal diseases that have yet to breakout. The lower rates of periodical vaccination intentions against a disease that is regarded as an on-going pandemic, as measured in the information provision condition (which is similar to official recommendations), may suggest that periodical pandemic-containing vaccines may be perceived as less effective and beneficial than periodical pandemic-preventing vaccines, and hence require external incentivization in order to enhance their perceived utility and reach similar acceptance rates. Despite its limitations, this study offers an important contribution to public policy literature and to policy makers in their efforts to explain and steer vaccination acceptance while facing an ongoing pandemic. Future research should explore the effects of other monetary and non-monetary types of incentives, as well as the interaction effect of incentive type and valence.

## Data Availability

The datasets generated during and/or analyzed during the current study are available from the corresponding author on reasonable request.

## Footnotes

1 Official data published by the Israeli Ministry of Health: https://datadashboard.health.gov.il/COVID-19/general.

2 Official data published by the Israeli Ministry of Health: https://www.health.gov.il/PublicationsFiles/Flu2019_2020.pdf.
